# Lymphocyte Subpopulations in the Healthy Human Lacrimal Gland and Their Variations With Age and Sex, Systematic Review 1960–2023

**DOI:** 10.1002/iid3.70167

**Published:** 2025-03-19

**Authors:** Claudia M. Trujillo‐Vargas, Luisa María Rendón‐Macías, Ronald Yamil Paredes Guerrero, Cinta S. de Paiva, Jaiberth Antonio Cardona‐Arias

**Affiliations:** ^1^ Grupo de Inmunodeficiencias Primarias, Facultad de Medicina, Universidad de Antioquia UdeA Medellín Colombia; ^2^ Escuela de Microbiología, Universidad de Antioquia UdeA Medellín Colombia; ^3^ Department of Ophthalmology Baylor College of Medicine Houston Texas USA

**Keywords:** aging, lacrimal gland, lymphocytic infiltration, lymphocytic subpopulations

## Abstract

**Background:**

Immunosenescence has been associated with an imbalance in the lacrimal functional unit and histopathological changes in exocrine glands, especially in women.

**Objective:**

To define the main lymphocyte subpopulations in the human lacrimal gland and their variations with age and sex, according to scientific articles published between 1960 and 2023.

**Methods:**

A systematic review was performed on PubMed, ScienceDirect, and Google Scholar. The PRISMA 2020 guidelines were applied for the search and selection of studies.

The methodological quality was evaluated with the STROBE guidelines. A meta‐analysis of three selected articles dichotomizing lymphocytic infiltrates according to age group was also performed.

**Results:**

We selected 20 observational studies, including 774 healthy individuals (722 cadavers). The articles evaluated the lymphocyte infiltration with hematoxylin and eosin staining, immunohistochemistry and immunofluorescence. There was high variability in the criteria to define the apparently human lacrimal and to quantify the lymphocytic infiltration. There was an underrepresentation of individuals younger than 40 years (12.6%), and female sex (38.9%). Three articles reported an association of age and sex with lymphocytic infiltration in the healthy lacrimal gland, while two articles did not. Plasma cells were the most abundant lymphocyte subpopulation in the healthy lacrimal gland, including IgA‐containing plasma cells. B cells were reported to be very scarce in the LG in two articles. In the meta‐analysis of three selected articles, no statistical difference in lymphocytic infiltration was found between individuals younger and older than 60.

**Conclusion:**

There is the need of further observational studies, better defining the study design, with similar representation across sex and ages to ascertain what are the changes of lymphocytic composition in the lacrimal gland related to age and sex. Further studies are also needed to assess the dynamics of lymphocytic populations in a more detailed manner using cutting‐edge methodologies such as single‐cell sequencing or transcriptomics.

**Trial Registration:**

PROSPERO 2023 CRD42023435653 Available from: https://www.crd.york.ac.uk/prospero/display_record.php?ID=CRD42023435653.

## Graphical abstract

The articles evaluated the lymphocyte infiltration with hematoxylin and eosin staining, immunohistochemistry and immunofluorescence. Plasma cells were the most abundant lymphocyte type in the healthy lacrimal gland, including IgA‐containing plasma cells. This research updates the evidence available in this field, generates hypotheses to guide subsequent studies and demonstrates that it is an under‐researched topic in immunology

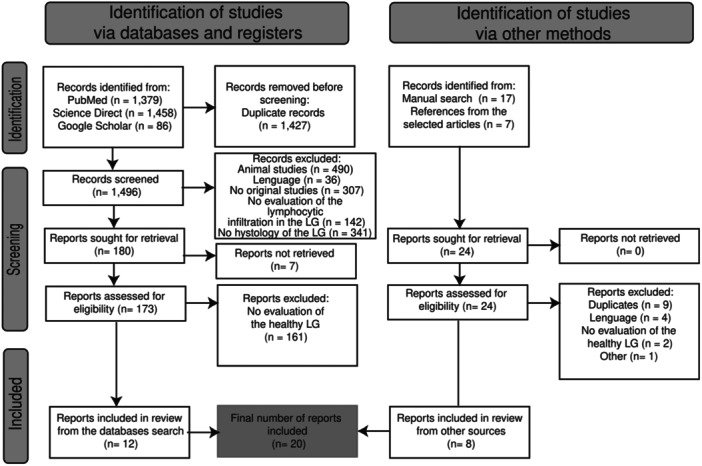

## 1 Introduction

The lacrimal functional unit (LFU) consists of the conjunctiva, cornea, meibomian, lacrimal glands, eyelids, and the nerves that innervate the mucosal surface of these structures in the LFU. This set of tissues maintains the integrity of the tear film at the ocular surface, protects the cornea and improves the quality of the retina image [1]. The lacrimal gland (LG) is an exocrine gland that produces the tear film aqueous component, made of water, proteins and electrolytes. This tubular organ consists of acini, ducts and myo‐epithelial cells. The ducts are organized in lobes supported by a matrix of connective tissue [2]. There are two types of LG: main and accessory [2]. The accessory LGs correspond to 10% of the total LG mass, with large interindividual variations [3, 4]. As part of the eye‐associated lymphoid tissue, cells and molecules from the immune system are also usual constituents of the LG [5].

Dysfunction of the components of the LFU cause keratoconjunctivitis sicca, or dry eye disease (DED) [1]. Although frequently underestimated, DED is a symptomatic condition producing blurred vision, sensitivity to light, burning, redness and roughness in the eye, and can cause corneal ulcers and vision loss. The symptomatic disease is more prevalent among elderly women than men of the same age, and has been considered one of the most common reasons for seeking clinical eye care [6, 7]. DED has a global prevalence of about 12% [6, 7]; it is estimated that DED will steadily increase in the coming decades due to our high dependency on digital screens since early age, a behavior that reduces eye blink frequency and increases the likelihood of ocular surface damage [8].

Several autoimmune diseases manifest in the LG and may cause DED [9]. Among them, Sjögren's syndrome (SS) is characterized by dysfunction of the exocrine glands (salivary and lacrimal gland, among others), causing dryness in eyes and mouth. SS is associated with an increase in effector lymphocytes migration into these tissues. Similar to DED, SS has a greater prevalence in women, and also in the elderly compared to middle‐age adults [10]. The higher prevalence of DED and SS in relation to age and sex suggests that these two factors may trigger pathophysiological processes resulting in LFU dysfunction and LG degeneration characterized by periductal fibrosis and atrophy [11].

Among the mechanisms that predispose women to autoimmune diseases, the role of sex hormones and chromosomes, microbiome and other environmental factors have been investigated [12]. On the other hand, immunosenescence, a phenomenon toward chronic inflammation associated with hyperactivation and exhaustion of the immune system, is another pathophysiological process related to aging [13]. Immunosenescence might be the key triggering factor of DED or even systemic autoimmune diseases that affect the ocular surface. It is postulated that abnormal infiltration of highly activated lymphocytes in the LG would lead to exaggerated recognition of autoantigens, resulting in tissue damage and defective homeostasis of the LFU [14]. In mice, LG exhibits histological and innervation changes with age that are associated with the accumulation of lipofuscin and inflammatory cells, and the decrease in the secretion of antimicrobial substances [15]. In humans, several studies have evaluated the magnitude of the lymphocytic infiltrates in the LG in individuals with various diseases that affect the ocular surface, using tissues from cadavers or biopsies [16, 17]. However, there have been no studies addressing lymphocytic infiltration in the healthy human LG in relation to age and sex. This may help to define whether immunosenescence is a key factor for DED or other genetic and environmental cues are more influential for the expressiveness of this condition. Moreover, studies analyzing the lymphocytic infiltration of the healthy human LG have been performed in very heterogeneous populations, using various methodologies, evaluating different cellular markers and presenting discrepant results [18, 19, 20]. For this reason, it is necessary to consolidate the information from these articles through a systematic review. Our study aimed to investigate the main lymphocyte subpopulations in the human lacrimal gland and their variations with age and sex, according to scientific articles published between 1960 and 2023. The above may be the foundation for future public health studies to prevent the development of DED or other associated diseases, to provide a healthy aging process in the ocular surface.

## 2 Methods

### 2.1 Type of Study

Systematic review following the PRISMA 2020 guidelines [21]. The protocol was previously registered in PROSPERO *(International prospective register of systematic reviews)* (CRD42023435653) on June 25, 2023.

### 2.2 Search Strategy and Selection Protocol

We used the thesaurus MeSH (*Medical subject headings*) to identify synonyms for the terms *lacrimal gland* and *lymphocytic infiltration*. To increase the search sensitivity, we also performed a *Pearl Harvest*, in which the following additional terms were identified: Dry Eye Disease, Dry Eye Syndrome, Keratoconjunctivitis Sicca, Sicca Syndrome, Sjögren Syndrome, Xerophthalmia and Dacryoadenitis. Healthy controls are commonly included as a reference in the studies of these conditions. After selection of the search terms, we applied the search in PubMed, Science Direct and Google Scholar as specified in Table 1. Also, the reference section of the selected articles was reviewed to identify other relevant articles for the study. Duplicates were eliminated manually using filters in an Excel database.

**Table 1:** Search syntax applied in the databases.

PubMed	Science direct	Google scholar
(Lacrimal gland [Title/Abstract]) AND (Lymphocytic infiltration [Title/Abstract])	Title, abstract, keywords: Lacrimal gland AND Lymphocytic infiltration	allintitle: Lacrimal gland AND Lymphocytic infiltration
(Lacrimal gland [Title/Abstract]) AND (Lymphocyte [Title/Abstract])	Title, abstract, keywords: Lacrimal gland AND Lymphocyte	allintitle: Lacrimal gland AND Lymphocyte
(Lacrimal gland [Title/Abstract]) AND (T‐lymphocyte [Title/Abstract])	Title, abstract, keywords: Lacrimal gland AND T‐lymphocyte	allintitle: Lacrimal gland AND T‐lymphocyte
(Lacrimal gland [Title/Abstract]) AND (B‐lymphocyte [Title/Abstract])	Title, abstract, keywords: Lacrimal gland AND B‐lymphocyte	allintitle: Lacrimal gland AND B‐lymphocyte
(Lacrimal gland [Title/Abstract]) AND (T‐cell [Title/Abstract])	Title, abstract, keywords: Lacrimal gland AND T‐cell	allintitle: Lacrimal gland AND T‐cell
(Lacrimal gland [Title/Abstract]) AND (B‐cell [Title/Abstract])	Title, abstract, keywords: Lacrimal gland AND B‐cell	allintitle: Lacrimal gland AND ‌B‐cell
(Lacrimal gland[Title/Abstract]) AND (leukocyte[Title/Abstract])	Title, abstract, keywords: Lacrimal gland AND Leukocyte	allintitle: Lacrimal gland AND Leukocyte
(Lacrimal gland [Title/Abstract]) AND (Mononuclear cell [Title/Abstract])	Title, abstract, keywords: Lacrimal gland AND Mononuclear	allintitle: Lacrimal gland AND Mononuclear cell
(Lacrimal gland [Title/Abstract]) AND (Plasma cell [Title/Abstract])	Title, abstract, keywords: Lacrimal gland AND Plasma cell	allintitle: Lacrimal gland AND Plasma cell
(Lacrimal gland[Title/Abstract]) AND (Flow cytometry[Title/Abstract])	Title, abstract, keywords: Lacrimal gland AND Flow cytometry	allintitle: Lacrimal gland AND Flow cytometry
(Lacrimal gland [Title/Abstract]) AND (Fluorescence activated cell sorting [Title/Abstract])	Title, abstract, keywords: Lacrimal gland AND Fluorescence activated cell sorting	allintitle: Lacrimal gland AND Fluorescence activated cell sorting
(Lacrimal gland [Title/Abstract]) AND (Dry eye disease [Title/Abstract])	Title, abstract, keywords: Lacrimal gland AND Dry eye disease	allintitle: Lacrimal gland AND Dry eye disease
(Lacrimal gland[Title/Abstract]) AND (Dry eye syndrome[Title/Abstract])	Title, abstract, keywords: Lacrimal gland AND ‌Dry eye syndrome	allintitle: Lacrimal gland AND Dry eye syndrome
(Lacrimal gland [Title/Abstract]) AND (Keratoconjunctivitis sicca [Title/Abstract])	Title, abstract, keywords: Lacrimal gland AND Keratoconjunctivitis sicca	allintitle: Lacrimal gland AND Keratoconjunctivitis sicca
(Lacrimal gland[Title/Abstract]) AND (Sicca syndrome[Title/Abstract])	Title, abstract, keywords: Lacrimal gland AND ‌Sicca syndrome	allintitle: Lacrimal gland AND Sicca syndrome
(Lacrimal gland[Title/Abstract]) AND (Sjögren Syndrome[Title/Abstract])	Title, abstract, keywords: Lacrimal gland AND Sjögren Syndrome	allintitle: Lacrimal gland AND Sjögren Syndrome
(Lacrimal gland [Title/Abstract]) AND (Xerophthalmia [Title/Abstract])	Title, abstract, keywords: Lacrimal gland AND ‌Xerophthalmia	allintitle: Lacrimal gland AND Xerophthalmia
(Lacrimal gland[Title/Abstract]) AND (Dacryoadenitis[Title/Abstract])	Title, abstract, keywords: Lacrimal gland AND ‌Dacryoadenitis	allintitle: Lacrimal gland AND Dacryoadenitis

### 2.3 Screening and Eligibility Criteria

The following inclusion criteria were applied: publications with search terms in the title, abstract or keywords, studies in English, Spanish or Portuguese, articles containing original results, studies in humans, studies with histologic evaluation of the LG and those that quantified lymphocytic infiltration in the healthy lacrimal gland. No time restrictions were applied to the search. Unavailable articles and studies that did not include the evaluation of the lymphocytic infiltration in the LG were excluded.

### 2.4 Data Extraction, Reproducibility, and Evaluation of the Methodological Quality

The following information was extracted from the selected articles: Title, authors, year, journal and country of publication, type of study, type of LG included (either main or accessory), number of healthy LGs included, number of LGs analyzed per individual, age and sex of the individuals included, type of sample (from either cadavers or biopsies), criteria used to define healthy individuals, methodologies, molecular targets and criteria used to quantify the infiltration and finally, results regarding the type of infiltrates, the location and the association with age and sex.

The search and selection of studies, and the data extraction were carried out by two researchers independently and discrepancies were resolved by consensus.

The methodological rigor of the selected studies was evaluated using the criteria of methods section of the Strengthening the Reporting of Observational studies in Epidemiology (STROBE) guide [22].

### 2.5 Information Analysis

Qualitative synthesis of the extracted variables was carried out. From the studies that reported the degree of infiltration according to the age group, we dichotomized the infiltration as mild or moderate‐severe and the age as younger or older of 60 years of age. A meta‐analysis for the odds ratios was conducted evaluating homogeneity with *Q* statistic and *I* ^2^; the sensitivity analysis was carried out with the influence graph, the evaluation of possible publication biases with the Begg statistic, and the general result was reported with the Forest Plot. The analyzes were carried out in SPSS 29.0, with a *p* < 0.05 considered significant.

## 3 Results

### 3.1 Selection of the Studies and Analysis of the Methodological Rigor

**Figure 1:**
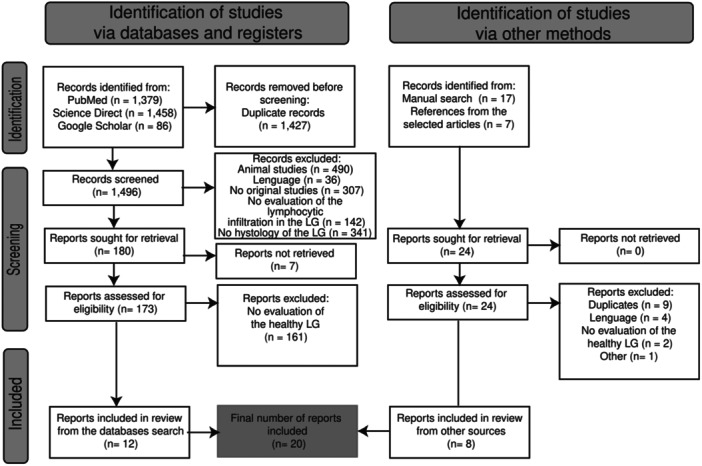
Flowchart for study selection according to the PRISMA 2020 guidelines [21]. *Source:* Own elaboration based on the included papers.

The search terms in the databases and in the manual search resulted in a list of 1496 and 24 articles for screening, respectively. From them, a total of 20 articles were included (Figure 1). The only article excluded in “others” was the article by Belfort et al., 1980 [23] because of the use of an obsolete methodology.

**Figure 2:**
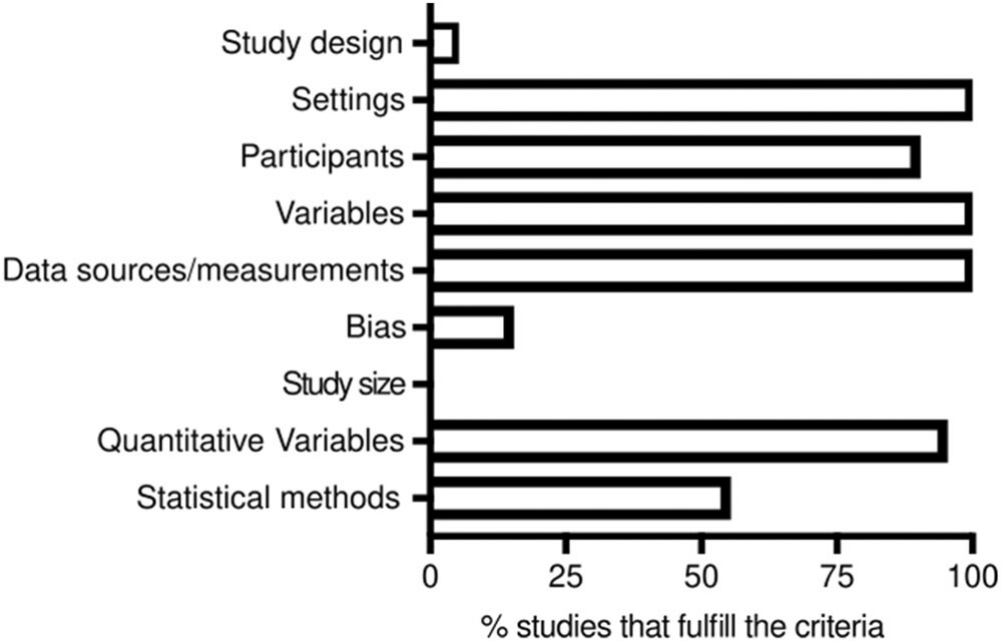
Evaluation of methodological quality according to the STROBE guidelines. *Source:*Own elaboration based on the included papers.

Because the 20 selected studies were classified as observational, the nine criteria from the methods section of the STROBE guidelines were applied to evaluate methodological rigor [22]. The results from this analysis are shown in Figure 2. Only Sato EA et al., 2010 specified the study design [24]. All the articles provided information about the study settings and data collection. From the 14 studies using cadaveric LG, only seven specified their collection by autopsy [4, 16, 18, 25, 26, 27, 28], others were collected either from an eye bank [29] or from LG excess tissues [30]. Five of the articles do not specify cadaver's origin [11, 19, 20, 31, 32]. From the eight papers that used LG from biopsies of living subjects, six specified their obtention during LG unrelated ocular surgeries [11, 17, 27, 33, 34, 35] and only in two studies, the biopsies were performed exclusively for the related study [24, 36]. We found information about the place and relevant dates of data collection only in eight [16, 18, 19, 25, 26, 30, 32, 36] and two articles [18, 33], respectively.

Two studies did not provide information about the eligibility criteria [28, 30]. In general, the remaining studies classified the healthy individual by the absence of autoimmune, systemic, connective tissue‐related or ocular diseases. Wieczorek R. et al. and Franklin RM et al. included patients with eye tumors, but not involving histologically the LG [17, 34].

Regarding the definition of variables, all studies specified how lymphocytic infiltration was evaluated. Only Roen JL et al. quantify qualitatively the infiltration (mild, moderate) [28]. All the studies specified the data sources and measurements, however, only 11 (55%) applied descriptive statistics or statistical methods in the analysis. Three studies (10%) describe the efforts to address potential sources of bias [11, 25, 26]. Lastly, none of the studies explain how the study size was calculated, presumably because the LG were included upon availability.

### 3.2 Description of the Selected Studies

Table 2 specifies the characteristics of the apparently healthy individuals used in each study. The 20 selected studies included 774 healthy individuals. Fourteen studies were classified as cross‐sectional and six were case‐control studies (Table 3).

**Table 2:** Characteristics of the healthy individuals/lacrimal glands (LG).

Reference	Characteristics of the healthy individuals/lacrimal glands (LG) included
Waterhouse JP, 1963 [16]	Free from neoplasm involving lymphocyte‐like cells, had not received a cytotoxic drug within recent months. LG were accepted if a representative section of 1 × 1 cm^2^ was available.
Franklin RM et al., 1973 [34]	Presumably normal LG. No evidence of a significant infectious process present by examination or by history.
Williamson J et al., 1973 [31]	No RA or connective tissue disease, no history of ocular disease.
Allansmith MR et al., 1976 [4]	Healthy volunteers, free of ocular complaints and normal eyes by examination.
Murray SB et al., 1981[20]	Subjects that did not die as a result of, or were suffering from, any connective tissue disease.
Damato BE et al., 1984 [11]	Patients randomly selected and cases were excluded if the quality or quantity of the material did not permit histological grading of the degree of fibrosis.
Nasu M et al., 1984 [18]	No affected with autoimmune diseases, leukemia, malignant lymphoma, or local tumor or infectious diseases in the orbit.
Allansmith MR et al., 1985 [27]	Cadaver's autopsies without eye disease or biopsies from patients undergoing ocular surgery for reasons unrelated to the lacrimal gland.
Gudmundsson OG et al., 1988 [19]	Free of eye or LG disease.
Wieczorek R et al., 1988 [17]	Individuals that underwent ocular operative procedures not involving histologically the LG.
Segerberg‐Konttinen M., 1989 [26]	Glands with pathological changes due to infection or neoplasm were not included. Gland lobules with duct dilatation caused by obstruction and gland tissue with extravascular polymorphonuclear leukocytes were discarded. Subjects with lymphoma or leukemia, a recent history of immunosuppressive therapy, visible autolytic changes, hematomas or other recent damage to the face or neck were excluded. Glands were accepted if they contained an adequate amount of representative tissue (1 cm^2^).
Pepose JS et al., 1990 [32]	Control glands with no history of ocular disease.
Obata H et al., 1995 [37]	Autopsies were excluded if the patients had autoimmune diseases (including SS), RA, endocrine diseases, head irradiation, amyotrophic lateral sclerosis and graft‐versus‐host disease.
Sato EA et al., 2010 [24]	None of the subjects had a history of ocular surgery, other ocular or systemic diseases, or a history of topical/systemic drug or contact lens use that would alter the ocular surface.
Wong AJ, et al., 2014[36]	Eyes with noninflamed orbits.

*Note:* Not specified in Mircheff A.K. et al., 1991 [30], Roen et al., 1985 [28], Dua H.S. et al., 1994 [29], Sacks E.H. et al., 1986 [33], Brandtzaeg P. et al., 1979 [35].

Abbreviations: DM, diabetes mellitus; LG, lacrimal gland; RA, rheumatoid arthritis; SS, Sjögren Syndrome.

**Table 3:** Characteristics of the studies and samples.

Reference	Study type	Number of individuals^a^	Type of LG	Sample type
Waterhouse J.P., 1963 [16]	Descriptive cross‐sectional	226	Main	Cadavers
Franklin R.M. et al., 1973 [34]	Descriptive cross‐sectional	6	Main	Biopsies
Williamson J. et al., 1973 [31]	Case‐control	10	Main	Cadavers
Allansmith M.R. et al., 1976 [4]	Descriptive cross‐sectional	10^b^	10 main and 10 accessory	Cadavers
Brandtzaeg P. et al., 1979 [35]	Case‐control	13	Main	Biopsies
Murray S.B. et al., 1981 [20]	Descriptive cross‐sectional	74	Main	Cadavers
Damato B.E. et al., 1984 [11]	Descriptive cross‐sectional	25	Main	23 cadavers and 2 biopsies
Nasu M. et al., 1984 [18]	Case‐control	115	Main	Cadavers
Allansmith M.R. et al., 1985 [27]	Descriptive cross‐sectional	12^d^	11 main and 1 accessory	5 cadavers and 7 biopsies
Roen J.L. et al., 1985 [28]	Descriptive cross‐sectional	32^c^, ^e^	Main	Cadavers
Sacks E.H. et al., 1986 [33]	Descriptive cross‐sectional	1	Accessory	Biopsies
Gudmundsson O.G. et al., 1988 [19]	Descriptive cross‐sectional	14	Main	Cadavers
Wieczorek R. et al., 1988 [17]	Descriptive cross‐sectional	7	Main	Biopsies
Segerberg‐Konttinen M., 1989 [26]	Descriptive cross‐sectional	102^f^	NS	Cadavers
Pepose J.S. et al., 1990 [32]	Case‐control	7	Main	Cadavers
Mircheff A.K. et al., 1991 [30]	Descriptive cross‐sectional	12	Main	Cadavers
Dua H.S. et al., 1994 [29]	Descriptive cross‐sectional	2	NS	Cadavers
Obata H. et al., 1995 [37]	Descriptive cross‐sectional	80	Main	Cadavers
Sato E.A. et al., 2010 [24]	Case‐control	9	Main	Biopsies
Wong A.J. et al., 2014 [36]	Case‐control	7	NS	Biopsies
**Total**	14 Descriptive cross‐sectional/6 case‐control studies	774	651 main/12 accessory/111 unknown	722 cadavers/52 biopsies

Abbreviations: DM, diabetes mellitus; LG, lacrimal gland; NS, nonspecified; RA, rheumatoid arthritis; SS, Sjögren Syndrome.

^a^ Only individuals classified as controls are considered.

^b^ Main and accessory.

^c^ Two LG (left and right) from the same individual are analyzed.

^d^ These studies combined the analyses of controls and a patient with SS.

^e^ Two patients with DM and one with RA.

^f^ Three patients with DM and one with RA.

Table 4 presents the age group's distribution in all the individuals with healthy LG in the eleven studies with available information. This represents 460/774 (59.4%) from the total population. We found a minor representation (12.6%) of individuals younger than 40 years whereas more than a quarter of the population belonged to the age group of 61‐70 years. It was not possible to extract the age group's distribution of the healthy individuals in nine of the selected studies (Table 4). Some studies did not provide the age of the participants [33, 34, 36]. The other studies presented the age data by using descriptive age statistics as follows: Waterhouse J.P., 1963 [16] 226 controls with an age range 0–75; Williamson J. et al., 1973 [31] 10 controls with an age range 32–76 and a mean of 64; Brandzaeg P. et al., 1979 [35] 13 controls with an age range 2–65; Pepose J.S. et al., 1990 [32] 7 controls with an age range 25–65; Sato EA et al., 2010 [24] 11 controls with a mean of 48.6 ± 14.9; Damato B.C. et al., 1984 [11] 97 controls with an age range 7–93 and a mean ± SD 62 ± 17.

**Table 4:** Age distribution of the individuals included.

Age groups	Studies with available information											Total (%)
	4	20	18^b^	27	28^b^, ^d^	19	17	26^e^	30	29	25^b^	
**0–10**	0	2	NS	0	0	0	1	0	0	1	2	**6 (1.3%)**
**11–20**	0	2	NS	1^c^	0	0	0	3	0	0	2	**8 (1.7%)**
**21–30**	0	1	NS	3	0	1	0	4	0	0	0	**9 (2.0%)**
**31–40**	0	1	17^a^	1	1	1	0	13	1	0	0	**35 (7.6%)**
**41–50**	4	5	13	1	4	2	1	18	0	0	12	**60 (13%)**
**51–60**	2	15	24	2	7	0	1	15	0	0	18	**8 (18.3%)**
**61–70**	2	19	32	3	12	5	2	17	1	0	28	**121 (26.3%)**
**71–80**	1	15	22	1	3	1	1	16	4	0	16	**80 (17.4%)**
**80** +	1	14	7	0	5	4	1	16	6	1	2	**57 (12.4%)**
**Total**	**10**	**74**	**115**	**12**	**32**	**14**	**7**	**102**	**12**	**2**	**80**	**460**

*Note:* Age in years.

Abbreviations: DM, diabetes mellitus; F, female; M, male; NS, nonspecified; RA, rheumatoid Arthritis; SS, Sjögren Syndrome.

^a^ The age group is 0–40.

^b^ The age groups are distributed as 0–39, 40–49, 50–59 and so forth.

^c^ Child (13 years old) with SS.

^d^ Include two men with DM (67 and 84 years old) and one woman with RA (85 years old).

^e^ Include three patients with DM (34 years old, M, 60 and 64 years old, F) and one man with RA (58 years old).

Table 5 specifies the sex per age group of the individuals in the six studies with available information (303/774, 39.1% of the population included). The female:male distribution in these studies was 38.9 versus 61.1% with a greater representation of males in the age group from 41 to 80+ years.

**Table 5:** Sex distribution of the healthy individuals included.

Age groups	Sex	Studies with available information						Total F	Total M	Total
		20	18	27	19	17	24			
0–10	F	1	NS	0	0	1	1	3		3
	M	1	NS	0	0	0	1		2	2
11–20	F	1	NS	0	0	0	1	2		2
	M	1	NS	1^c^	0	0	1		3	3
21–30	F	1	NS	2	0	0	0	3		3
	M	0	NS	1	1	0	0		2	2
31–40	F	1	9^a^	0	1	0	0	11		11
	M	0	8	1	0	0	0		9	9
41–50	F	3	6	0	1	1	5	16		16
	M	2	7	1	1	0	7		18	18
51–60	F	8	7	0	0	1	6	22		22
	M	7	17	2	0	0	12		38	38
61–70	F	7	15	1	2	2	8	35		35
	M	12	17	2	4	0	20		55	55
71–80	F	3	6	0	0	1	3	13		13
	M	12	16	1	1	0	13		43	43
80+	F	8	0	0	3	1	1	13		13
	M	6	7	0	1	0	1		15	15
**Total**		**74**	**115**	**12**	**15**	**7**	**80**	**118**	**185**	**303**

*Note:* ^a^The age group is 0–40; ^b^The age groups are distributed as 0–39, 40–49, 50–59 and so forth; ^c^Child (13 years old) with SS.

Abbreviations: F, female; M, Male; NS, nonspecified.

### 3.3 Methods of Evaluating the Lymphocytic Infiltration of the LG

In the 20 selected studies, we found three methodologies to evaluate lymphocytic infiltration: hematoxylin‐eosin staining (H&E), immunohistochemistry (IHC), and indirect immunofluorescence (IF). Twelve studies evaluated lymphocytic infiltration by H&E [4, 11, 16, 17, 18, 20, 24, 25, 26, 28, 31, 36]. However, the quantification was very heterogeneous. Four studies [16, 18, 25, 26] used the term “focus” for quantification, defined as an aggregate of 50 or more cells. However, Wieczorek R. et al. [17] defined focus as an aggregate of 30 cells or more. Only Segerberg‐Konttinen M. used the term *Focus score*, defined as the number of foci in a 4‐mm^2^ area of gland tissue [26]. Waterhouse JP quantified foci/section in 1‐cm^2^ of lacrimal tissue [16]. Murray S.B. et al [20] neither defined the “focal” aggregates nor the atypical “focal” inflammatory cell infiltrate. On the other hand, Roen J.L. et al., Damato BE et al and Segerberg‐Konttinen M. measured infiltration through dichotomous variables whereas Sato E.A. et al. calculated the inflammatory cell density per mm^2^ [11, 24, 26, 28]. Only Williamson J. et al. classified the lymphocytic infiltration in stages and also took into account the structural changes in the gland [31]. Two studies quantified plasma cells in the LG by H&E [27, 36]. We illustrated in Table 6 the great variability in the studies that measure lymphocytic infiltration severity by grades or stages.

**Table 6:** Studies that measure the lymphocyte infiltration in the LG by severity using the haematoxylin & eosin staining.

Reference	Measurement Unit	Asigned stage					
		0	1	2	3	4	5
Waterhouse J.P., 1963 [16]	Grades. A foci defined as an aggregate containing more than 50 cells.	0–1 focus per section. No infiltration.	2–8 foci per section. Slight infiltration.	9–40 foci per section. Moderate infiltration.	> 40 foci per section, Severe infiltration.	More than half the gland parenchyma is replaced in a section.	NS
Williamson J. et al., 1973 [31]	Stages	Normal LG. Plasma cells in the Connective tissue; lymphocytes less frequently detected.	Mild chronic inflammation. Abnormal arrangements of the ducts, slight intralo	Severe chronic inflammation. Total destruction of the normal lobular pattern, dense infiltration with lymphocytes, aggregating into lymph follicles, and progressive acinar atrophy.	Late fibrosis. Fibrous tissue is increased. Few acinar cells remain.	NS	NS
Murray S.B. et al., 1981 [20]	Grades	Scattered plasma cells located around the acini and throughout the fibrous septa.	Lymphocytes commonly seen around the ductules	Lymphocytes as focal aggregates within the lobules and next to the central ducts. Grading according to density.	Lymphocytes as focal aggregates within the lobules and next to the central ducts. Grading according to density.	Lymphocytes as focal aggregates within the lobules and next to the central ducts. Grading according to density.	Striking atypical focal inflammatory cell infiltrate.
Nasu M. et al., 1984 [18]	Grades. Each focus consists of 50 or more lymphocytes or plasma cells.	No cellular infiltration	One or two foci. Very slight infiltration.	More than three foci (multiple) in all sections.	Multiple foci or diffuse lymphocytic infiltration in all sections with mildly destroyed tissue.	Multiple foci or diffuse lymphocytic infiltration in all sections with moderately or severely destroyed acinar tissue.	NS
Obata H. et al., 1995 [37]	Grades. Lymphocytic foci are defined as 50 or more cells.	Not present per section.	One focus per section.	Two or more foci per section.	NS	NS	NS

Abbreviations: LG, lacrimal gland; NS, nonspecified.

As shown in Tables 7 and 8, we also found great variability in the quantification of lymphocytic or plasma infiltration in the LG, respectively, using the different methodologies.

**Table 7:** Immunohistochemistry studies to evaluate lymphocytic infiltration in the lacrimal gland using specific markers.

Reference	Antibody clone (type of lymphocyte)	Criteria to quantify infiltration
Sacks E.H. et al., 1986 [33]	Leu3a/b (T CD4^+^); OKT8 (T CD8^+^); BLI/OKB2/Leu14 (B cells); OKT10 (Plasma cells)	Qualitative
Gudmundsson O.G. et al., 1988 [19]	Leu1 y Leu4 (Pan‐T); Leu3a/3b (T CD4^+^); Leu2a (T CD8^+^)	Cells/field (0.086 mm^2^ at 500×)
Wieczorek R. et al., 1988 [17]	OKM1, LeuMl (Leukocytes); OKT3 (Pan‐T); OKT4 (T CD4^+^), OKT8 (T CD8^+^); BL9 (Pan‐B); OKT10 (Plasma cells)	Cells/mm^2^
Pepose J.S. et al., 1990 [32]	Leu3 (T CD4^+^); Leu2 (T CD8^+^); BL9 (Pan‐B); OKB7 (B cells)	Percentages of the total number of infiltrating cells
Mircheff A.K. et al., 1991 [30]	Leu3 (T CD4^+^); Leu2 (T CD8^+^); Leu14 (B cells)	Cells/mm^3^
Dua H.S. et al., 1994 [29]	ICHT1 (T CD3^+^), OKT4 (T CD4^+^), OKT8 (T CD8^+^), CD22 (B cells)	Proportion of one marker in relation to the others

**Table 8:** Studies evaluating the immunoglobulin‐expressing plasma cells in the lacrimal gland.

Reference	Methodology	Antibodies	Criteria to quantify infiltration
Franklin R.M. et al., 1973 [34]	IF	Anti‐IgA, ‐G, ‐E, ‐M	Plasma cells/field at 500×
Allansmith M.R. et al., 1976 [4]	H&E, IF	Antibodies against the five Ig isotypes	Plasma cells/100 acini; crosses for Ig expression.
Brandtzaeg P. et al., 1979 [35]	IHC	Polyclonal IgG against the different Ig isotypes.	Percentages of the specific Ig‐expressing plasma cells in relation to the others.
Damato B.E. et al., 1984 [11]	H&E, IHC	NS	Plasma cells/0.09 mm^2^
Allansmith M.R. et al., 1985 [27]	IF	Monoclonal antibodies anti‐IgA, ‐IgA1, ‐IgA2	Percentages of IgA1 versus IgA2
Wong A.J. et al., 2014 [36]	H&E, IHC	Antibodies anti‐IgG and ‐IgG4	Cells/high power filed (400X, 0.3 mm^2^)

Abbreviations: H&E, Haematoxylin&Eosin Haematoxylin& Eosin staining; IF, immunofluorescence; IHC, Immunohistochemistry; Ig, Immunoglobulin; NS, Non specified.

### 3.4 Quantification of the Lymphocytic Infiltration in the Lg from Healthy Individuals

**Figure 3:**
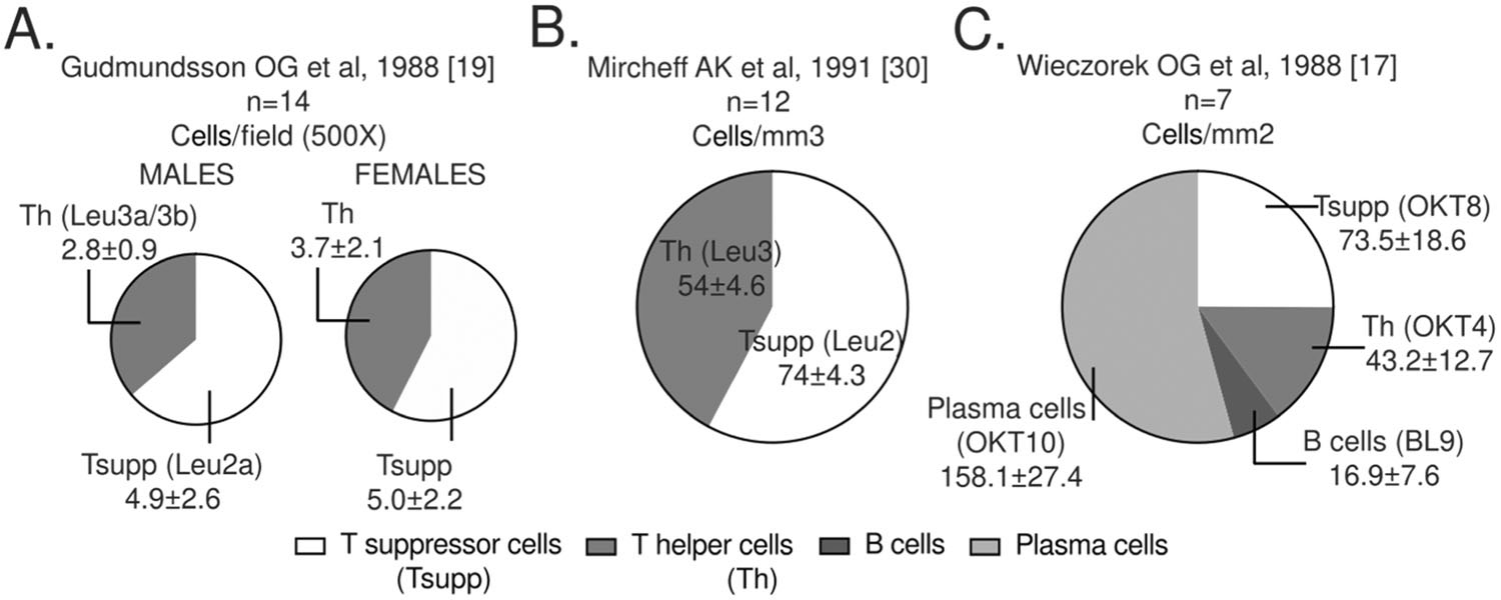
Distribution of lymphocytic subpopulations in the healthy human lacrimal gland. *Source:* own elaboration based on the included papers. The antibody clones used in each study are indicated in parentheses. Note that the measurement units in the three studies are different and are reported with the statistical mean ± standard error. LTh: Helper T lymphocytes, LTsup: Suppressor T lymphocytes, LB: B lymphocytes..

Putting together the information regarding the main lymphocyte subpopulations in the healthy human LG may help to define criteria for the normality of immune cell infiltration as well as to assess the role of the immune system in DED and related conditions. Moreover, these studies may help to corroborate the information obtained from animal models. From the studies with available data, Sato EA et al, 2010 [24], reported the inflammatory cell density of 377 ± 152 cells/mm^2^ in nine LG, a value significantly lower to that obtained from SS patients. The three IHC studies that quantified general lymphocyte subpopulations in the gland used different measurements (Table 7) (Figure 3) [17, 19, 30]. A consistent finding in these studies was the greater proportion of suppressor or cytotoxic (CD8 + ) as compared with helper T cells (CD4 + ) in the LG. This finding is also confirmed by Pepose JJ et al [32] who estimated the average of CD4 + T cells in 5‐25% of total number of infiltrating cells in the seven control glands analyzed whereas CD8 + T cells represented 25‐50%. Also, Dua HS et al, 1994 [29] described that CD8+ are twice as many as CD4 + T cells in the LG. Gudmundsson OG et al [19] compared the T cell proportion by sex and observed a slight but not significant increase CD4 + T cell absolute counts in females. The CD4:CD8 ratio in the LG from females and males reported in the later study was 0.74 and 0.57, respectively [19]. However, Wieczorek et al reported a CD4:CD8 ratio of 0.56 in the seven female LG analyzed [17].

Together with the CD4+ and CD8 + T cell values, Wieczorek et al calculated the absolute counts of B and plasma cells in the seven female LG [17]. Plasma cells were the most abundant lymphocytes in the gland (158 cells/mm^3^ or 53.9% of the total average mononuclear cells, (Figure 3C), a finding only qualitatively confirmed by Sacks EH et al [33] in an accessory LG of Krause from a superior forniceal specimen. These authors reported abundant plasma cells (stained with the OKT10 antibody, that target CD38) in the interstitium, between the acini of the glandular tissue, admixed with helper and suppressor T cells. B cells represented only a small proportion of the lymphocytes present in the human LG according to Wieczorek et al [17] (Figure 3C). However, Sacks EH et al [33] did not detect B cells in the accessory LG analyzed, by using three different monoclonal antibodies.

**Figure 4:**
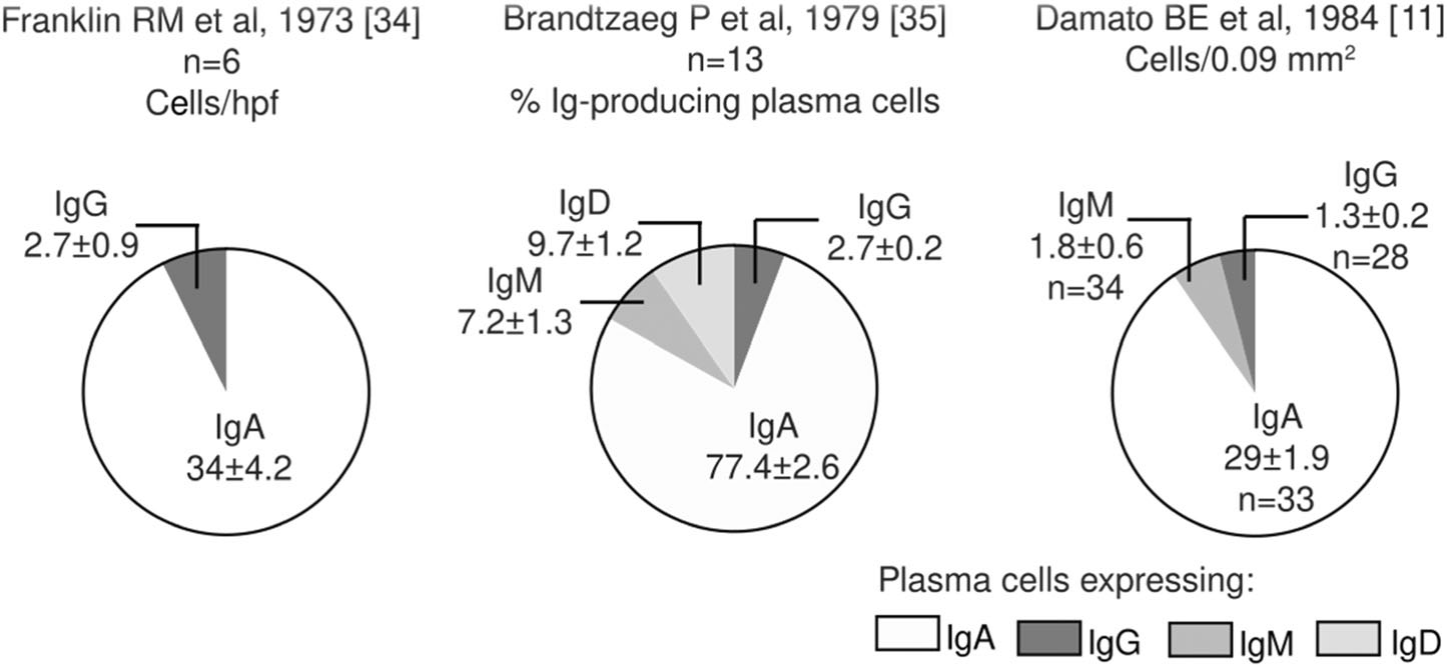
Distribution of Immunoglobulin (Ig)‐producing plasma cells in the healthy human lacrimal gland. *Source:* own elaboration based on the included papers. The data in the graph are presented with mean ± standard error.

Figure 4 shows the distribution of the Ig‐expressing plasma cells in the healthy human LG in the studies with available information. IgA‐expressing plasma cells are the most abundant, followed by those expressing IgD, IgG, IgM and IgE [11, 34, 35]. Allansmith et al [27] reported the IF plasma cell patterns per 100 acini in accessory LGs, however, their results lacked sensitivity. Wong AJ et al, 2014 [36] reported IgG4‐expressing plasma cell counts of less than 10 per high power field in seven controls. Other interesting data was presented by Allansmith MR et al [27], who observed greater plasma cell counts in the main LG as compared to the accessory gland and conjunctiva, despite the fact that the latter is the tissue most exposed to the external environment. They also reported an average count of 188 IgA1/IgA2+ cells/section (range: 58‐356) [27]. Another report from the same author [4] described the average percentages of IgA1‐ versus IgA2‐expressing plasma cells in the LG (56 *vs.* 44%), with no difference between the 10 main and one accessory glands from controls compared to one from a SS patient. Wieczorek R et al, 1988 reported that kappa predominates over lambda light chains in a proportion of 2:1 [17].

### 3.5 Distribution of the Lymphocytic Infiltration in the LG from Healthy Individuals

In contrast to the information about the lymphocytic infiltrate counts and percentages in the LG from the selected studies, the evidence regarding the distribution of the different lymphocyte subpopulations was very consistent. Pepose JS et al observed T helper cells scattered within the interstitium, predominantly at the periphery of the lymphocytic foci. Only a few cytotoxic‐suppressor T cells were scattered throughout lymphoid aggregates, however, this was the predominant phenotype in the interstitium, far from the lymphoid aggregates and interspersed between acinar cells. B cells were found in the center of primary follicles and in occasional secondary follicles [32]. A similar distribution had previously been reported by Wieczorek R et al who also compared CD4+ versus CD8 + T cell counts specifically in the lymphoid aggregates and primary follicles from five of seven glands. The CD4:CD8 average ratio in this location was 2.2. They also reported that only 16% of the CD4 + T cells preferred glands or ducts to the interstitium [17]. Dua HS et al also reported suppressor CD8 + T cells in the proximity of the acini and close to the epithelial lining of the ducts [29]. Gudmundsson OG et al described small to medium sized T‐lymphocytes in the interacinar tissue, often adjacent to an acinus or close to a collecting duct [19].

Regarding Ig‐expressing plasma cells, Murray SB et al observed them scattered around the acini and throughout the fibrous septa [20]. Franklin RM et al reported increased numbers of IgA‐expressing plasma cells in the interstitium [34], a finding validated by Wieczorek R et al [17] and also by Sacks EH et al [33] in an accessory LG.

Obata H et al [37] calculated the percentages of lymphocytic foci and periductal lymphocytic infiltration as evaluated by H&E, in the palpebral vs the orbital LG lobes. In LG with two or more lymphocytic foci per section, the orbital lobe exhibited significantly more lymphocytic foci.

### 3.6 The Association of the Lymphocytic Infiltration with Age and Sex

The association of the lymphocytic infiltration with age and sex specified in the selected articles is summarized in Table 9. There was no association between the Ig‐producing plasma cell counts or distribution, with age and sex in three studies [4, 11, 27]. By using H&E, Waterhouse JP and Nasu M et al observed significantly more lymphocytic infiltration in women older than 45 years or individuals older than 40 years, respectively [16, 18]. However, two other studies did not report this association [19, 20].

**Table 9:** Association of the lymphocytic infiltration in the lacrimal gland with age and sex.

Reference	Statistical Test	Type of association and results
Waterhouse JP, 1963 [16]	χ^2^ with Yate's correction, Mann‐Whitney U‐test	Significantly greater focal adenitis in women of all ages than in men and higher in women over 45 years than in all other subjects.
Murray SB et al, 1981 [20]	NS	In all but four specimens, the inflammatory cell content was uniform throughout the age range.
Damato BE et al, 1984 [11]	χ^2^ and Mann‐Whitney U‐tests	None of the plasma cell populations showed significant variation with age and sex.
Nasu M et al, 1984 [18]	χ^2^ test	There was no sex difference in the prevalence of lymphocytic infiltration.
		The prevalence of lymphocytic infiltration was low in younger groups and high in older groups. There was a significant difference between those who were older than 40 and those younger than 40.
Allansmith MR et al, 1985 [27]	NS	The distribution and number of IgAl‐ or IgA2‐containing cells were not associated with age and sex.
Gudmundsson OG et al, 1988 [19]	Mann‐Whitney U‐test	The distribution of the T‐lymphocytes seemed relatively even, and no obvious, difference in appearance, location, or distribution of the T‐cells was detected according either to the age or sex of the studied subjects or to the various T‐cell subsets. Statistical analysis did not reveal any sex related difference in the T‐cell density.

NS: H&E, Haematoxylin‐eosine staining; IF, immunofluorescence; IHC, Immunohistochemistry non‐specified.

Therefore, we collected the available information and dichotomized the data about lymphocytic quantification into null/mild or moderate/severe per group age, to perform a meta‐analysis. Only in three of the selected studies it was possible to retrieve this information [18, 26, 28] from a total of 249 individuals (32% of the LG from the selected articles). The dichotomization was performed as follows: In Nasu M et al, grade zero or no infiltration was classified as null/mild and the other grades as moderate/severe [18]. In Roen JL et al, no infiltration was classified as null/mild and the presence of inflammatory cells (in 15 of the studied LG) was classified as moderate/severe [28]. In Segerberg‐Konttinen we considered a focus score of less/equal or greater as 1 as either null/mild or moderate/severe infiltration, respectively [26]. Waterhouse JP and Murray SB et al only presented the data about the lymphocytic infiltration in the LG by age group in figures and therefore, were excluded from this meta‐analysis [16, 20]. The analyses of null/mild versus moderate/severe lymphocyte infiltration per age group was only possible between individuals either younger or older than 60 years and showed no statistical difference. We neither observed statistical heterogeneity nor a publication bias among the studies. Roen and Segerberg‐Konttinen include patients with AR and DM (five and two, respectively) that were not possible to separate in the analyses [26, 28].

**Figure 5:**
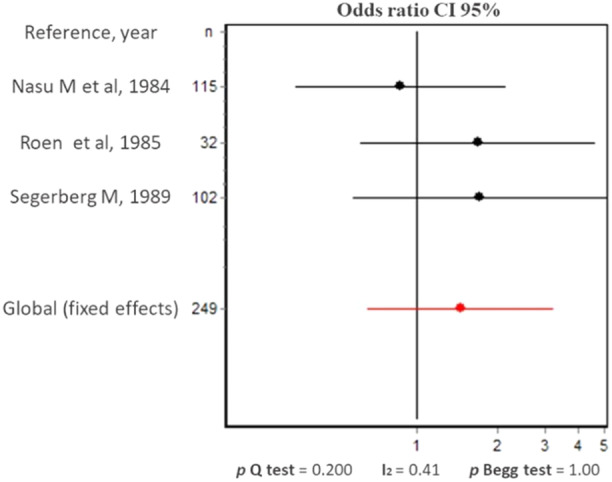
Forest plot of the comparison of lymphocytic infiltration in the lacrimal gland by age (< 60 vs. ≥ 60 years old). *Source:* Own elaboration based on the included papers.

The combined results of the meta‐analysis showed no statistical differences between the degree of infiltration and the two age groups given that the odds ratio combined (with a random effects model) was 1.5 (CI 95% 0.7; 3.2) (Figure 5).

## 4 Discussion

LFU dysfunction that manifests in DED is more frequently observed in elderly women. Lymphocyte infiltration in the LG has been postulated as the triggering factor for this condition, in association with glandular tissue atrophy. There is a plethora of information in the literature about the lymphocyte infiltration of the LG in animal models and humans, however, no one has systematically collected and analyzed the published data from human samples. In the present study, we aimed to systematically review the information related to the lymphocyte subpopulations in the healthy human LG and analyze its association with age and sex. It was possible to retrieve twenty observational studies that fulfilled the criteria of eligibility. The selected studies included 774 healthy individuals. Among the selected articles, the lack of studies using updated methodologies to evaluate lymphocyte infiltration in the LG is outstanding. Although H&E, IHC and IF are still useful tools for this purpose, high‐throughput sequencing technologies, advanced flow cytometry and confocal microscopy techniques constitute promising tools to define the different subpopulations of T and B cells (memory, activated, resident, regulatory, among others) present in the human LG and their associated molecular markers [38].

The first goal of our systematic review was to compile the information regarding the lymphocyte subpopulations in the healthy human LG. Two of the selected papers showed that plasma cells were the most abundant lymphocytes in the normal gland, representing more than half of the mononuclear cells [17, 33]. Among them, IgA‐containing plasma cells were the most abundant with almost equal ratio IgA1/IgA2 and 2:1 usage of kappa vs lambda chains [17, 33]. Similar results have been found in healthy major and minor salivary glands, observing also a predominance of IgA+ plasma cells in the interstitium followed by the presence of other lymphocytes both scattered among the interstitium and in aggregates close to the ducts [39]. The later study also failed to detect B cells in the majority of the healthy salivary glands, a finding that was also reported in the healthy LG in one of the selected articles from the present review [33]. The preponderance of plasma cells has also been reported for other mucosal surfaces, namely periodontal tissue although others revealed predominant memory B cells compared to naïve and antibody‐secreting cells in clinically healthy gingiva [40]. In deep tissues such as the lung, the immune landscape seems to differ, with greater quantities of myeloid, T and NK cells, although an immune niche for IgA plasma cells at the airway submucosal glands prevailed [41].

A very consistent finding among the selected papers was the preponderance of cytotoxic/suppressor CD8+ over helper CD4 + T cells among the healthy LG. These cells were also distributed differentially with CD4 + T cells preferentially located at the periphery of the lymphocytic foci whereas CD8 + T were more prevalent in the interstitium. Since memory T cells are more frequent at the mucosal surfaces, a plausible explanation for the altered ratio CD4:CD8 in the healthy LG as compared to blood is the documented faster rates of cell division of CD8+ as compared to CD4+ memory T cells which are associated with lower stimulation requirement and TCR activation threshold [42]. However, in the normal human intestinal tract the percentage of T‐helper cells vs suppressor cells is quite different depending on location in the mucosa, according to Gudmundsson [19].

The crucial questions would be: (1) What is the role of these immune cells in the healthy LG, (2) What are their antigen‐specificities and (3) Whether their variations are clinically relevant. Sato E.A. et al. reported a three‐fold increase in the density of inflammatory cells in the LG from SS patients compared to controls, associated with decreased acinar unit density and diameter [24]. Allansmith M.R. et al. reported similar percentages of IgA1‐ versus IgA2‐expressing plasma cells in controls as compared with one SS patient [27]. Pepose J.S. et al. reported increased frequency of B and T‐helper cells in the SS LG with few cytotoxic suppressor T cells [32]. Nasu found increased severity in the lymphocyte infiltration in the LG of patients with several autoimmune diseases as compared to individuals without autoimmune diseases [18].

Regarding the relationship of the lymphocyte infiltration with age and sex in the LG, Waterhouse J.P. and Nasu M. et al. observed significantly more lymphocytic infiltration in women older than 45 years or individuals older than 40 years, respectively [16, 18]. Nevertheless, two other studies did not report this association [19, 20]. A meta‐analysis of three of the selected studies with available information about lymphocyte infiltration per age group (32% of the total individuals from the selected articles) [18, 26, 28] did not show statistical increase in lymphocyte infiltration among individuals older than 60 years compared to the younger ones.

In summary, this systematic review found a higher representation of plasma cells followed by CD8+ cytotoxic/suppressor T lymphocytes, CD4+ helper T lymphocytes and B cells in the healthy lacrimal gland. Most of these plasma cells expressed IgA. A meta‐analysis was conducted with three studies, indicating that lymphocytic infiltration in the lacrimal gland may not undergo significant changes in healthy individuals after 60 years of age. The most significant limitation of the published studies about the lymphocyte infiltration in the human healthy LG is related to the structural data heterogeneity in sample selection, detection methods and quantification standards. This is precisely what justifies this systematic review. The low percentage of women and people under 40 may contribute to a lack of representativeness, which could lead to findings that do not accurately reflect the circumstances in various age and gender categories. Although the meta‐analysis found no significant heterogeneity, it does not rule out unreported bias. There was also a lack of information about the health status and lifestyle choices of the LG donors, likely associated with the difficulties to find LG live donors or cadavers. The results of our meta‐analysis should be interpreted with caution because the inflammatory changes in the LG related to age may likely start at middle adulthood [16, 18] but this information needs to be validated. These limitations represent a challenge to harmonize criteria and consolidate information in future studies. It also becomes necessary to implement cutting‐edge molecular and cellular techniques to elucidate the different subpopulations of lymphocytes present in the lacrimal gland and understand the factors determining infiltration.

## Author Contributions

**Claudia M. Trujillo‐Vargas:** conceptualization, data curation, formal analysis, funding acquisition, investigation, methodology, project administration, resources, software, supervision, validation, visualization, writing – original draft, writing – review and editing. **Luisa María Rendón Macías:** conceptualization, data curation, formal analysis, investigation, methodology, software, validation, visualization, writing – original draft, writing – review and editing. **Ronald Yamil Paredes Guerrero:** conceptualization, data curation, formal analysis, investigation, methodology, software, validation, visualization, writing – original draft, writing – review and editing. **Cinta S. Paiva:** conceptualization, funding acquisition, methodology, supervision, validation, visualization, writing – original draft, writing – review and editing. **Jaiberth Antonio Cardona‐Arias:** conceptualization, formal analysis, funding acquisition, investigation, methodology, software, supervision, validation, visualization, writing – original draft, writing – review and editing.

## Ethics Statement

The authors have nothing to report.

## Consent

The authors have nothing to report.

## Conflicts of Interest

The authors declare no conflicts of interest.

## Data Availability

All relevant data supporting the conclusions of this article are included within the article. Any additional information is available from the corresponding author upon reasonable request.
